# Measuring predictability of autonomous network transitions into bursting dynamics

**DOI:** 10.1186/1471-2202-15-S1-P2

**Published:** 2014-07-21

**Authors:** Sima Mofakham, Michal Zochowski

**Affiliations:** 1 Department of Biophysics, University of Michigan, Ann Arbor, MI, USA; 2 Department of Physics, University of Michigan, Ann Arbor, MI, USA

## 

Understanding spontaneous transitions between dynamical modes in the neuronal networks is of great importance. For example, such transitions separate pathological and normal function of the brain. In this study, we developed a metric to capture the characteristics of such spontaneous transitions from asynchronous to synchronous dynamics and predict occurrence of these transitions in a simplified neuronal network. In this work we simulate excitatory and excitatory-inhibitory network and in these neuronal networks we examine how inhibition, network topology, noise level, and cellular heterogeneity affect network dynamics. In case of excitatory network, we saw three different types of dynamics depending on its underlying structure: 1) local connectivity leads to long propagating waves of activity, 2) small-world connectivity results in two coexisting phases of short waves of activity and episodes of synchronous activity, and 3) random connectivity pattern that provides synchronous dynamics. When the connectivity pattern is within the small-world region (Pe=0.1-0.2), firing rate is optimum and transitions into and out of the synchronous activity are frequent. Here noise is another factor that can shape dynamics, as our results indicate substantial increase in the noise level can prevent occurring of spontaneous transitions from asynchronous to synchronous dynamics.

Then, we investigated how various topologies of inhibitory connectivity affect the network’s spatio-temporal patterning. When the excitatory network has a fixed small world connectivity pattern (P_e_=0.15), local inhibitory connectivity pattern significantly suppresses propagating chains of activity, even though the firing rate does not change remarkably. However, when inhibitory cells are connected randomly to other inhibitory and excitatory cells, network dynamics adopts long local traveling chains of activity with low frequency and the synchronous activity of the network was suppressed completely. Interestingly we learned that for a fixed connectivity pattern of excitatory network, when inhibition topology is in small-world region the probability that network goes under synchronous activity is maximized compare to the local and random connectivity pattern.

Ultimately, we developed and utilized different measures based on the spike timings to detect early sings of possible transitions into the bursting regime. These measures go through systematic changes prior to the onset of transitions into the bursting that can be interpreted as the early signs of incoming transition and based on that we can obtain a lead-time to predict future transitions. We show that this lead-time is a function of connectivity pattern of excitatory and inhibitory cells, and our predictability is higher when inhibitory neurons have small-world connectivity pattern rather than solely local or random connectivity.

